# The Aroma Fingerprints and Discrimination Analysis of Shiitake Mushrooms from Three Different Drying Conditions by GC-IMS, GC-MS and DSA

**DOI:** 10.3390/foods10122991

**Published:** 2021-12-03

**Authors:** Dong Chen, Lei Qin, Yue Geng, Qinglong Kong, Silu Wang, Songyi Lin

**Affiliations:** National Engineering Research Center of Seafood, School of Food Science and Technology, Dalian Polytechnic University, Dalian 116034, China; chendong689689@126.com (D.C.); qinlei@dlpu.edu.cn (L.Q.); gy15242464088@163.com (Y.G.); KongQingLong0420@163.com (Q.K.); wangsl2021@163.com (S.W.)

**Keywords:** aroma fingerprints, discrimination analysis, gas chromatography-ion mobility spectrometry (GC-IMS), descriptive sensory analysis (DSA), advanced chemometric methods

## Abstract

The aroma fingerprints and discrimination analysis of shiitake mushrooms under different drying conditions were performed by GC-IMS, GC-MS, and descriptive sensory analysis (DSA) with advanced chemometric methods. Three samples (A, B, and C) were treated with varied drying degree and rate. The sample A and C were at the same drying degree and the sample B and C were at the same drying rate. The GC-IMS volatile fingerprints, including the three-dimensional topographic map, topographic map, and gallery plot, showed that 29 compounds showed higher signal intensities in sample B. Moreover, 28 volatile compounds were identified by HS-SPME-GC-MS and only 8 compounds were ever detected by GC-IMS. The sample B not only had more varieties of volatile compounds, but also showed significant higher contents than sample A and C, especially C8 compounds (*p* < 0.05). Additionally, sample B showed the highest intensity in mushroom-like, chocolate-like, caramel, sweat, seasoning-like, and cooked potato-like odors by DSA. PCA, fingerprint similarity analysis (FSA) and PLSR further demonstrated that the sample B was different from sample A and C. These results revealed that samples with different drying degree were different and drying degree exerted more influence on the volatile flavor quality than the drying rate. This study will provide a foundation and establish a set of comprehensive and objective methods for further flavor analysis.

## 1. Introduction

Shiitake mushrooms (*Lentinus edodes*), belonging to fungi phylum, tricholoma, are the second most cultivated edible mushrooms in the world and the production in China accounts for 80% of the world’s production [1,2]. The popularity of shiitake mushrooms may be attributed to the abundant nutrients, including bioactive polysaccharides, essential amino acids, poly-unsaturated fatty acids, ergosterol, vitamin C, folate, niacin, and minerals [3,4]. These nutrient substances gain the shiitake mushrooms remarkable properties, such as decreasing the risk of hypertension, hypercholesterolemia and cancer, supporting the human immune response, and enhancing human resistance to the common cold and other diseases [5,6,7]. Furthermore, shiitake mushrooms are highly favored by consumers worldwide owing to the unique flavor, especially in northeast Asian, and commonly serve as a delicacy on the table or used as food-flavoring materials in dishes [8,9].

Fresh shiitake mushrooms are prone to undergo postharvest quality deterioration, such as weight loss, browning, nutrient loss, and rotting, which lead to their short storage and reduce their value. Drying is the most important and most commonly used method for preservation of agricultural foods, which reduces the moisture content to a safety level, inhibits microbiological, enzymatic, and chemical degradations, and reduces transportation costs [10,11]. Now, hot air drying (HAD) becomes a widely used method in mushroom processing industry, due to its low cost, easy control, short process time, and uniformity and hygiene for industrial food applications [7,12].

Flavor, as an important quality indicator of products, has gained more attention from manufacturers and researchers. Comparative studies on the flavor of shiitake mushrooms processed by HAD and other dried methods have been carried out in recent years. Tian et al. [7] compared the effects of HAD, vacuum drying, microwave drying, and microwave vacuum drying on volatile compounds of shiitake mushrooms. Wang, Li, Han, Ni, Zhao, and Hao [13] conducted comparative analysis on volatile compounds of dried shiitake mushrooms using HAD, infrared drying, and intermittent microwave coupled with hot air drying. Hou et al. [14] investigated the flavor profiles and flavor-related compounds of HAD, compared with vacuum freeze drying and the combined drying. Additionally, Qin et al. [15] studied the aroma profile of shiitake mushrooms during HAD through volatile compounds and sensory analysis. Chen, Wang, Li, Hao and Lin [16] reported that C8 compounds (including 1-octen-3-ol, 3-octanol, 2-octen-1-ol, 1-octanol, octanal, 2-octenal, and 3-octanone) showed significant decline (*p* < 0.05) during the early stage of hot air drying, while the sulfur compounds (including the thioether compounds and cyclic sulfur compounds) increased during the later stage, either in pileus or stipes of shiitake mushrooms. However, few researches focused on the effects of different drying rate and drying degree on the flavor of shiitake mushrooms.

Gas chromatography-mass spectrometry (GC-MS) is a matured analytical mass spectrometry technique and regarded to be the “gold standard” for the analysis of volatile flavor and fragrance compounds. It can combine with headspace solid-phase microextraction (HS-SPME) in food analysis [17,18]. HS-SPME-GC-MS integrates the safety and accuracy of HS-SPME, high separation ability of GC, and the superiority of mass spectrometry in the identification of substances [19]. This allows it to be applied in qualitative and quantitative analysis of volatile components and provide details of compounds in food analysis. However, it is a time-consuming operation [20,21].

Gas chromatography-ion mobility spectrometry (GC-IMS) is an emerging mass spectrometry technique and is gaining increasing popularity, with automatic headspace sampling (HS) for the analysis of volatile flavor compounds [18]. When a mixture of volatile compounds is analyzed using GC-IMS, the analytes are first separated by the GC (retention time) and then the elution is separated in the ion mobility spectrometer (drift time) at atmospheric pressure, thereby producing a multidimensional spectrum including retention time, drift time, and signal strength [21,22]. In addition, it can be used to distinguish between isobaric compounds and certain isomers, which usually are co-eluted from the GC column and hardly be distinguished by mass spectrometry, as well as distinguish the samples with nontargeted volatile fingerprints [23,24]. Compared with GC-MS, GC-IMS has advantages such as intuitive visualization of data, high separation efficiency, high analysis speed, operation at atmospheric pressure, inexpensive maintenance, and possible portability [18,25]. It can be applied in the establishment of volatile flavor fingerprints for food classification and adulteration detection, evaluation of food freshness and spoilage, detection of the off-flavor of food products, monitoring volatile metabolites during food processing, changes in volatile components during storage [26]. For instance, GC-IMS was applied to develop the flavor fingerprint of Tricholoma matsutake Singer and detect that the concentration of C8 compounds, including 3-octanone, 3-octanol, 1-octen-3-one, and 1-octanol, decreased after drying [25]. Moreover, the characteristic volatiles fingerprints of different parts of fresh and dried Tricholoma matsutake Singer were established by HS-GC-IMS and the formation of some volatile compounds (hexanal, heptanal, 2(5H)-furanone, acetophenone, nonanal, benzeneacetaldehyde) were observed after drying [27]. GC-IMS was also used to reveal that HAD caused considerable losses of volatile compounds except for volatile acids, while vacuum freeze-drying and combined drying preserved aroma compounds effectively [14]. However, GC-IMS is unable to replace the GC-MS. The lack of a library-like NIST mass spectral library of GC-MS and the non-linear response of IMS hider the qualitative and quantification of GC-IMS in flavor analysis [26,28].

Therefore, GC-MS or GC-IMS could not be determined as the best analytical technique. However, either technique could be used as a complementary technique for the other one [29]. More comprehensive information of volatile compounds could be revealed and discrimination of food samples could be conducted by the combination of GC-MS and GC-IMS [27]. The collaborative analysis has been applied in a few studies in order to establish the characteristic volatiles fingerprints and investigate the volatile compounds among of Tricholoma matsutake Singer [27], finger citron [30], kiwifruit [21], white and yellow rice [17], and virgin olive oil [29]. The combination of GC-MS and GC-IMS is a hot topic and continued trend. Nonetheless, the combination of the two mass spectrometry methods is rarely performed regarding the flavor fingerprint and volatiles of shiitake mushrooms.

Furthermore, aroma perception could not be provided only by chromatography analysis [14]. Descriptive sensory analysis (DSA) is an effective tool to detect and describe the qualitative and quantitative sensory attributes of products, and to determine the association of product characteristics and consumer perception [21,31]. Quantifying the magnitude of the predominant sensory descriptors of shiitake mushroom by different drying methods has been reported by Politowicz et al. [10]. However, little is documented in literature about the effects of different drying rate and drying degree on the aroma properties of shiitake mushrooms.

Thus, the objective of this study is to develop a comprehensive flavor fingerprint, to investigate the effects of drying rate and degree on aroma profile of shiitake mushrooms, to conduct the discrimination analysis of shiitake mushrooms under different drying conditions by the combination of GC-MS, GC-IMS, and DSA with advanced chemometric methods.

## 2. Materials and Methods

### 2.1. Materials

Fresh shiitake mushrooms (L-808, cultivated in Northeast China) were purchased from a local market in Dalian, Liaoning province, China. Shiitake mushrooms with similar size and without mechanical damage were picked out. After removing the surface dirt and the stipes, the samples were processed by HAD. The initial moisture contents of the samples were 88.82% on the wet basis (w. b.).

### 2.2. HAD Processing

The samples were divided into three identical groups and spread out a single layer on the tray. Samples were processed by HAD, at 60 °C in a constant-temperature drying oven (DHG-9053A, Jinghong laboratory equipment Co., Shanghai, China) with an air flow speed of 1 m/s and an air humidity of 10%.

Each pileus from the first group was cut into four equal parts. The first group was processed by HAD until the weight reached constant. The drying time was 10 h. The processed first group was the sample A.

Each pileus of the second group was intact, without cutting. The second group was processed by HAD for 10 h. The weight of this group did not reach constant. The processed second group was the sample B.

Each pileus of the third group was intact, without cutting. The third group was processed by HAD until the weight of this group reached constant. The drying time was 14 h. The processed third group was the sample C.

After drying, samples were transferred to a desiccator with silica gel to cool down for 0.5 h to room temperature. After cooling, samples were stored at −80 °C kept in sealed aluminum foil bags until further analyses.

### 2.3. Measure of Moisture Content and Drying Rate

During drying, the moisture loss of samples was measured using the electronic analytical balance (BS224S, Sartorius, Germany) by an hour at early stage and then more frequently until drying process was terminated. The moisture content on wet basis was measured by the gravimetric method (GB/T5009.3-2016, National Standard of China). Shiitake mushroom samples were dried at 105 °C in oven until the constant weight, moved to a desiccator for cooling, and then weighed using the electronic analytical balance to obtain the dry weight. Moisture content on wet basis (*X*_n_) was calculated as Equation (1)

*X*_n_ = (*m*_n_ − *m*_o_)/*m*_n_ (1)

where *X*_n_ referred to the moisture content on wet basis (%) at n hour, n referred to the drying time in hours, *m*_n_ was the weight of material after drying for n hours, and *m*_o_ was the dry weight of the material.

### 2.4. Measure of Drying Rate

Drying rate (*V*) was calculated as Equation (2) according to Ozcelik, Ambros, Heigl, Dachmann, and Kulozik [32]

*V* = (*m*_n−1_ − *m*_n_)/(*t*_n_ − *t*_n−1_) (2)

where *m*_n−1_ and *m*_n_ were the weight measured between two sequential times, while (*t*_n_ and *t*_n−1_ represented the corresponding time. Drying rate was expressed as %/h.

### 2.5. HS-GC-IMS Analysis

HS-GC-IMS analysis was performed as described by Li et al. [25] with some modifications. Analyses of shiitake mushrooms were completed on a combined device of an Agilent 490 gas chromatograph (Agilent Technologies, Palo Alto, CA, USA) using a FS-SE-54-CB-1 capillary column (15 m × 0.53 mm × 1.0 μm; 5% phenyl.95% dimethyl polysiloxane; CS-Chromatographie Service GmbH, Germany) and IMS instrument (FlavourSpec^®^, Gesellschaft für Analytische Sensorsysteme mbH, Dortmund, Germany), equipped with an autosampler unit (CTC Analytics AG, Zwingen, Switzerland) that can be directly sampled from the headspace by using a 1 mL air-tight heated syringe.

For analysis, samples were treated with WK-1000A micronizer (Jingcheng Medical Equipment Manufacturing Co., Ltd., Qingzhou, China). The shiitake mushroom powder (1 g) was weighed and placed into a 20 mL headspace glass sampling vial. Subsequently, samples were incubated at 40 °C for 25 min. After incubation, 500 µL of headspace was automatically injected into the injector (80 °C, splitless mode) by means of a heated syringe at 50 °C. Then the samples were driven into a FS-SE-54-CB capillary column (40 °C isothermal conditions) by nitrogen at a programmed flow as follows: 2 mL/min for 2 min, 30 mL/min for 8 min, 100 mL/min for 10 min, 150 mL/min for 5 min. The analytes were driven to the ionization chamber to be ionized in a positive ion mode by a 3H ionization source with 300 MBq activity. The resulting ions were driven to the drift tube (9.8 cm in length) which operated on a constant temperature (45 °C) and voltage (5 kV).

The drift gas (nitrogen gas) was set at 150 mL/min. Each spectrum had an average of 12 scans. All analyses were performed in triplicate. Nketones C4-C9 (Sinopharm Chemical Reagent Beijing Co., Ltd., Beijing, China) were used as external references to calculate the retention index (RI) of volatile compounds. Volatile compounds were identified by comparing RI and the drift time (the time it takes for ions to reach the collector through drift tube, in milliseconds) of standard in the GC–IMS library.

### 2.6. HS-SPME-GC-MS Analysis

HS-SPME-GC-MS analysis was performed as described by Guo et al. [27] with some modifications. Powder samples (0.8 g) with saturated NaCl solution (4 mL) were hermetically sealed in a 20 mL headspace glass having a PTFE/silicon septum (Supelco, Bellefonte, PA, USA) and a magnetic screw cap. Then the samples were incubated at 40 °C for 40 min. A stainless-steel needle containing divinylbenzene-carboxen-polydimethylsiloxane (DVB/CAR/PDMS, 50/30 μm) SPME fiber (Supelco, Bellefonte, PA, USA) was inserted through the septum of the sample vial to extract the volatile compounds at 40 °C for 40 min.

The volatile compounds were determined by the Agilent 7890B/5977B GC-MS instrument (Agilent Technologies Inc., Palo Alto, CA, USA) with the VF-WAXms capillary column (30 m × 0.25 mm × 0.25 μm, Agilent Technologies Inc., Palo Alto, CA, USA). The sample extracted by HS-SPME was injected in splitless mode, with the inlet at 250 °C. The carrier gas was 99.999% pure helium at a fixed flow rate of 1.68 mL/min. The initial oven temperature was 35 °C, ramped at 2 °C/min to 130 °C, then increased to 250 °C at 8 °C/min and held for 10 min. Mass detector was conducted in an electron impact mode at 70 eV and the ion source temperature was 230 °C. Mass spectra (MS) were scanned from 50 to 550 amu. The retention index (RI) of each compound was calculated using n-alkanes C7–C30 (Sigma-Aldrich, St. Louis, MO, USA) as external references. The RIs were calculated according to the retention time of n-alkanes. The volatile compounds were identified both by comparing RIs with those of the authentic compounds and by matching the mass spectra in the standard NIST 14 library. The reverse match factors of identified compounds were bigger than 900, whose RI value differed within 20 from those in the database. Volatile compounds were approximately quantitated according to the Equation (3) by adding 50 μL, 50 mg/mL cyclohexanone as the internal standard [33]. The concentration of the volatile compound in the shiitake mushrooms samples was calculated according to the Equation (4).

(3) $$\frac{m_{\mathrm{is}}}{m_{\mathrm{tc}}}=\frac{A_{\mathrm{is}}}{A_{\mathrm{tc}}}$$

(4) $$c=\frac{m_{\mathrm{tc}}}{0.8}$$

In Equation (3), *m*_is_ and *m*_tc_ were the concentrations of internal standard and target compound in the mixture of powder samples and NaCl solution, respectively, *A*_is_ and *A*_tc_ were the peak areas of internal standard and target compound, respectively.

In Equation (4), *c* was the concentration of the volatile compound in the shiitake mushrooms samples and expressed by µg/g.

### 2.7. Descriptive Sensory Analysis (DSA)

DSA was carried out as described by Zhao et al. [21] and Zhang, Lao, Bi, Pan, and Wu [34], including the procedures of panel selection, descriptors development, panel training, and sample evaluation. Aroma was determined by 20 experienced panelists (10 female and 10 male, 25 years old on average), who were trained and recruited from School of Food Science and Technology at Dalian Polytechnic University. The judgements of the panelists were averaged and got 70% accuracy through the sensory ability tests. Broken dried shiitake mushrooms powder (2 g) was presented in covered and odorless plastic vessels at room temperature. Seven attributes (mushroom, sweat, chocolate, caramel, sulfury, seasoning, and cooked potato) were selected for assessing samples. The assessors were asked to evaluate the intensities of selected seven characteristic aroma attributes. The intensities were ranged on a six-point scale from 0 (not perceivable) over 1, 2, ..., to 5 (strongly perceivable). The descriptors were compared with aqueous solutions of the following reference odorants: mushroom (1-octen-3-ol, the concentrations of 0, 1.50 × 10^−2^, 1.50 × 10^−1^, 1.50, 1.50 × 10, 1.50 × 10^2^ µg/g for the values of 0 to 5), sweat (isovaleric acid, the concentrations of 0, 44.10 × 3^−2^, 44.10 × 3^−1^, 44.10, 44.10 × 3, 44.10 × 3^2^ µg/g for the values of 0 to 5), chocolate (3-methyl-butanal, the concentrations of 0, 1.10 × 10^−2^, 1.10 × 10^−1^, 1.10, 1.10 × 10, 1.10 × 10^2^ µg/g for the values of 0 to 5), caramel (4-hydroxy-2,5-dimethyl-3(2H)-furanone, the concentrations of 0, 10.00 × 5^−2^, 10.00 × 5^−1^, 10.00, 10.00 × 5, 10.00 × 5^2^ µg/g for the values of 0 to 5), sulfury (lenthionine, the concentrations of 0, 0.27 × 10^−2^, 0.27 × 10^−1^, 0.27, 0.27 × 10, 0.27 × 10^2^ µg/g for the values of 0 to 5), seasoning (3-hydroxy-4,5-dimethyl-2(5H)-furanone, the concentrations of 0, 0.49 × 10^−2^, 0.49 × 10^−1^, 0.49, 0.49 × 10, 0.49 × 10^2^ µg/g for the values of 0 to 5), and cooked potato-like (3-(methylthio)-1-propanal, the concentrations of 0, 0.43 × 10^−2^, 0.43 × 10^−1^, 0.43, 0.43 × 10, 0.43 × 10^2^ µg/g for the values of 0 to 5).

### 2.8. Statistical Analysis

All experiments were performed in triplicate. GC-IMS assisted analysis software includes a VOCal with four plug-ins (reporter module, gallery plot module, dynamic PCA module, fingerprint similarity analysis module) and a GC × IMS library search function to analyze samples from different perspectives. IMS data were acquired and processed using VOCal processing software. Principal component analysis (PCA) was a multivariate statistical analysis technique to determine principal component factors instead of complex variables in the original samples. The PCA was performed through the normalization of the signal intensities to a range from 0 to 100 by VOCal. Fingerprint similarity analysis (FSA) is a measurement performed by VOCal that will be selected based on the Euclidian distance of the evaluation area intensities, containing all of the signal intensities. The x-shift represents the relative distance. The idea of FSA is a visual grouping/clustering based on similarities and highlights potential grouping/clustering. The partial least squares regression (PLSR) analysis was performed by SIMCA 14.1 software (Umeå, Sweden). Analysis of variance (ANOVA) was used to evaluate the differences between samples by the SPSS Statistic 22.0 software (SPSS Inc., Chicago, IL, USA), and *p* < 0.05 indicated statistically significant differences. Data were reported as the mean ± standard deviation.

## 3. Results and Discussion

**Figure 1:**
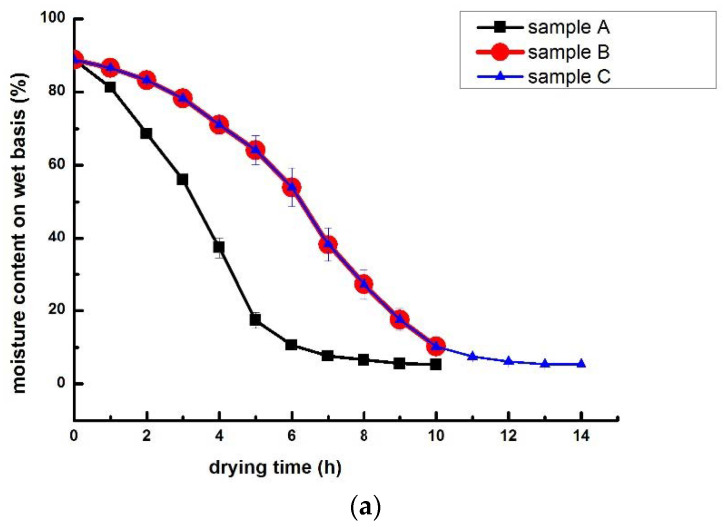

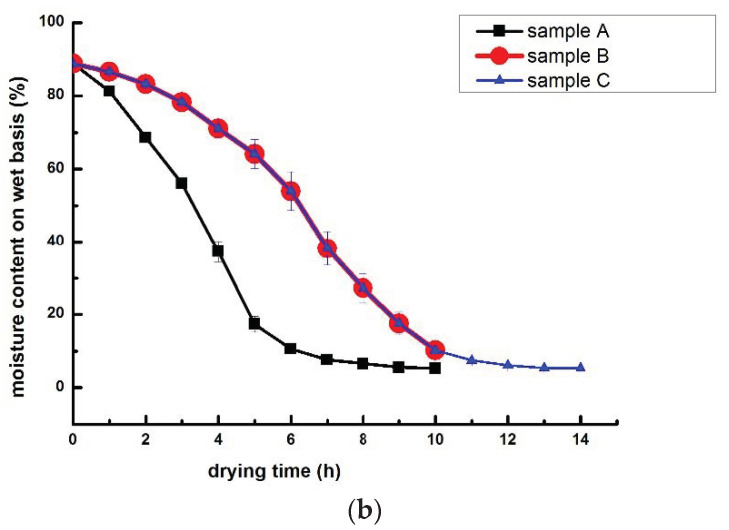
The moisture content (**a**) and drying rate curves (**b**) of shiitake mushrooms during hot air drying.

The moisture content and drying rate curves of samples during HAD were shown in Figure 1. The sample A and C reached the same drying degree (constant weight), but they were at the different drying rate. The sample B and C were at the same drying rate, but they were at different dying degree. The final moisture contents of sample A, sample B and sample C were 5.24%, 10.22% and 5.33%, respectively, meeting the national food safety standard (13%) for dried edible fungi and its products (GB7096-2014).

### 3.1. The Volatile Fingerprints of Shiitake Mushrooms under Different HAD Processing by HS-GC-IMS

**Figure 2:**
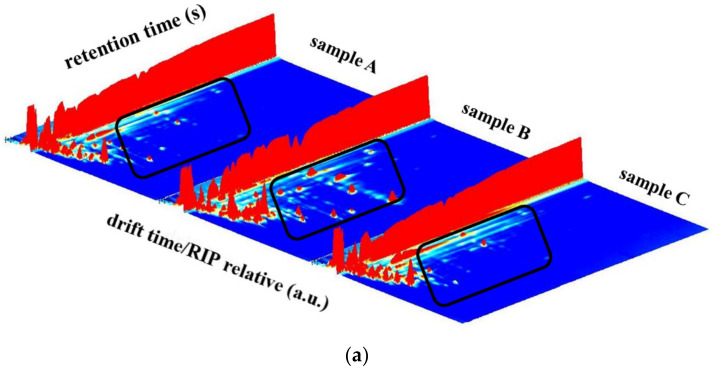

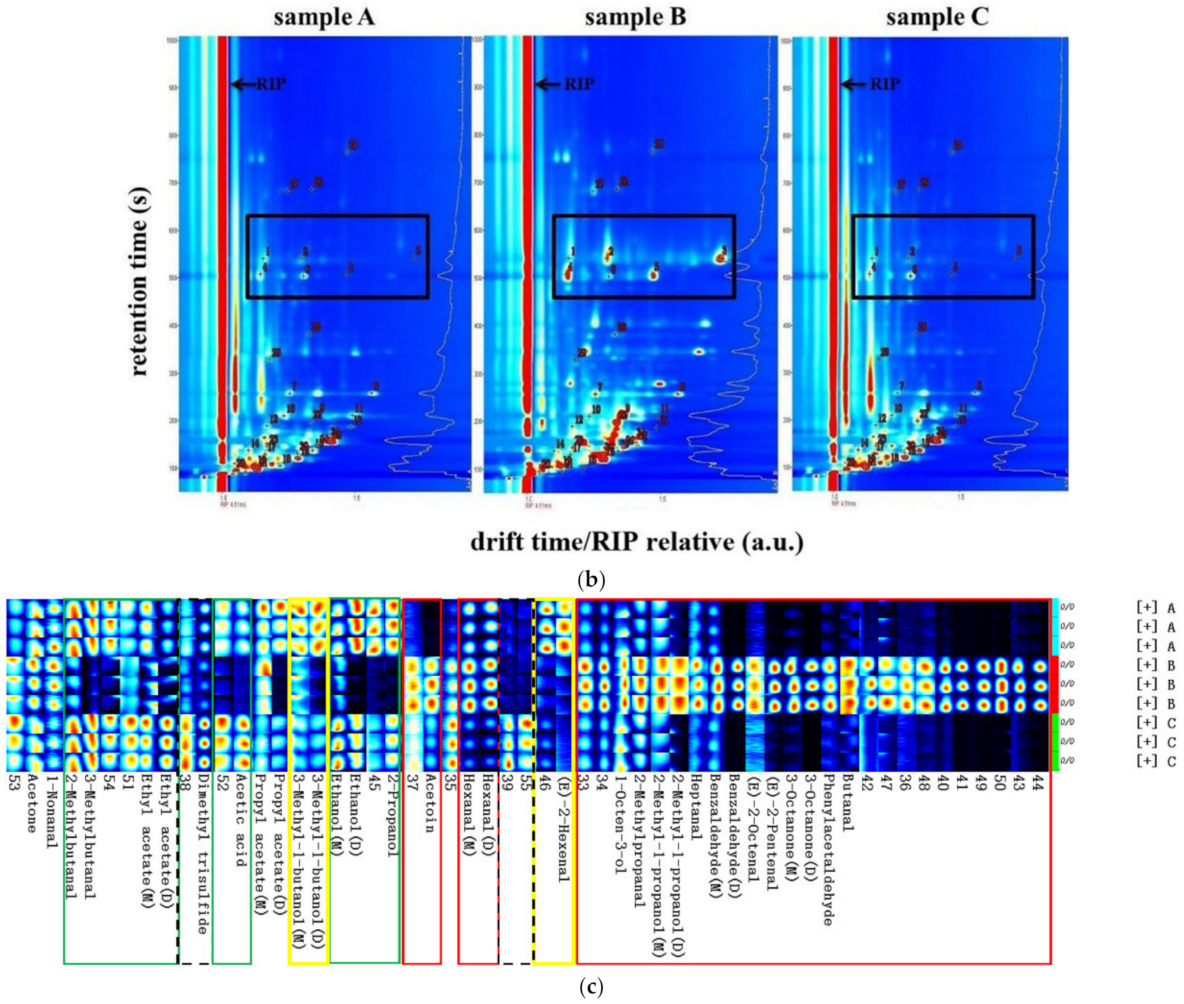
The fingerprints of volatile flavor compounds of shiitake mushrooms under different hot air drying conditions determined by GC-IMS: (**a**) 3D topographic plot, (**b**) topographic plot, and (**c**) gallery plot (The number in the Figure refers to the number in Table 1).

The GC-IMS technology was used to develop the fingerprints (Figure 2) and visually analyze the differences in the volatile components of shiitake mushrooms processed at different drying rates and different drying degree. The three-dimensional (3D) topographic map of the volatile components was presented in Figure 2a, with the drift time as the X-axis, the retention time as the Y-axis, and the peak intensity as the Z-axis. It could be seen that the varieties of volatile compounds in the three samples were similar, but the signal intensities were different. The content of volatile substances in sample B (shown in the black rectangle) was significantly higher than those in sample A and C.

Then to clearly show the different signal intensity, the GC-IMS topographic map was obtained (Figure 2b) through the top view of 3D topographic plot and by normalizing the ion migration time and the position of the reactive ion peak (RIP), using the relative drift time as the abscissa and the volatile matter retention time as the ordinate. It presented a global volatile fingerprint with 2D plots. Each point on the right of RIP represented a volatile organic compound. It could be seen that the retention time of most volatile compounds in all samples was 100–600 s, and the relative drift time was 1.0–1.5. Varieties of the volatile compounds in the three samples were also similar, as shown in 3D topographic map. The same substance might produce multiple signal points, including dimers and trimmers. This also was observed in the GC-IMS topographic maps of Tricholoma Matsutake Singer [25,27]. The color of the signal point represented the concentration of the substance, white indicated lower density, red indicated higher density, and darker colors indicated greater density [21]. It was showed that signal intensities of sample B were higher than those in sample A and C, especially those in the black rectangle. This indicated that the contents of volatile compounds in sample B were distinct from sample A and C, although they were similar with respect to the varieties of volatile compounds.

In order to effectively recognize discrepancy information between the three samples, all signals in topographic map were identified. A total of 55 volatile components were identified in the three samples, including 32 known components (Table 1) and 23 unknown components based on the existing database. Among the 32 known components, aldehydes, esters, alcohols, ketones, acids, and sulfur compounds were the main volatile flavor components.

All of the 55 signals were used to develop a gallery plot to compare the differential volatile compound information of the three samples (Figure 2c). Each row in the gallery plot was represented as all the signal peaks selected in a sample, and each column was represented as the signal peaks of the same volatile substance in different samples. From the Figure 2c, it could be seen that few compounds show similarity among the three samples. Among the 55 components, 29 components (in the red rectangle), including acetoin, hexanal, (E)-2-hexenal, 1-octen-3-ol, 2-methylpropanal, 2-methyl-1-propanol, heptanal, benzaldehyde, (E)-2-octenal, (E)-2-pentenal, 3-octanone, phenylacetaldehyde, butanal, showed obviously higher concentrations in sample B than those in sample A and C, whereas these 29 components showed similar levels between sample A and C. Moreover, the concentrations of 12 components (in the green rectangle) in sample B, including 2-methylbutanal, 3-methylbutanal, ethyl acetate, acetic acid, ethanol, 2-propanol, were markedly lower than those in sample A and C, and sample A were close to sample C with respect to these seven components. Additionally, four components (in the yellow rectangle) in sample A exhibited the highest concentrations and four components (in the black dashed rectangle) in sample C displayed the most abundant in contents among the three samples. Overall, the gallery plot demonstrated that sample B was obviously different from sample A and C, while sample A and C were similar compared with sample B. It might be due to the fact that the different drying extent between sample B and sample A and C led to different reactions and a differential reaction process. Chen et al. [16] has reported that volatile compounds in shiitake mushrooms changed significantly when drying progress approached to the constant weight. The volatile fingerprints obtained by GC-IMS revealed that samples with different drying degree were different and drying degree exerted more influence on the volatile flavor quality than the drying rate.

### 3.2. The Volatile Profile of Shiitake Mushrooms under Different Hot Air Drying Conditions by CG-MS

In general, GC-IMS analysis was a more sensitive detection of trace substances and obtained fingerprint information, but the focus of GC-IMS analysis was not to identify a single volatile compound [35]. Hence, the volatile compounds were comprehensively analyzed by GC-MS in this study. A total of 28 volatile compounds were identified by GC-MS combined with HS-SPME in the three shiitake mushroom samples, shown in Table 2. Eight compounds were detected by both GC-MS and GC-IMS, including 1-octen-3-ol, 3-octanone, benzaldehyde, dimethyl trisulfide, hexanal, acetic acid, phenylacetaldehyde, and 3-methylbutanal. The difference in the identified volatile components by GC-MS and GC-IMS might be due to the different working conditions and focal points of the two detection methods [36]. GC-MS was performed under phase heating and vacuum conditions and focused on qualitative and quantitative accuracy of substances, while GC-IMS was performed under constant temperature and atmospheric pressure and more focused on distinguishing samples [18,36]. This also stated the necessity of the combination of GC-IMS and GC-MS for the flavor analysis.

Among the 28 volatile compounds identified by GC-MS, 23 compounds, 24 compounds, and 20 compounds were identified, and the total contents of the identified volatile compounds were 41.02 µg/g, 70.18 µg/g and 53.83 µg/g, in sample A, B and C, respectively. The sample B not only had more varieties of volatile compounds, but also showed significant higher abundance in volatile compounds than sample A and C (*p* < 0.05). This stated that lower dryness degree would enrich the volatile flavor.

Sulfur compounds and C8 compounds have been reported to be the main volatile compounds in shiitake mushrooms, and sulfur compounds contributed the characteristic flavor of dried shiitake mushrooms and C8 compounds related to the fresh mushroom odor [37,38,39]. There were seven sulfur compounds, including carbon disulfide, dimethyl disulfide, thiovanic acid, dimethyl trisulfide, 1,2,4-trithiolane, 1,2,4,5-tetrathiane, and 1,2,4,6-tetrathiepane, which were identified in the three samples by GC-MS analysis. The total concentration of sulfur compounds in sample A was 24.63 µg/g, significantly lower than those in sample B (38.18 µg/g) and sample C (33.63 µg/g) (*p* < 0.05). Considering that sample A was at the higher drying rate than sample B and C, this result might indicate that higher drying rate led to lower content of sulfur compounds. Sulfur-containing compounds were mainly formed by sulfur-containing amino acids through Maillard reaction [7,15,40]. Different drying rate caused varied severities of reactions, which led to the dissimilarities in the contents of sulfur compounds among samples.

There were only three C8 compounds, including 1-octene-3-ol, 3-octanone and phenylethyl alcohol, which were identified in the three samples by GC-MS analysis. It was due to the fact that the drying process caused the oxidation and decomposition of C8 compounds [16,27]. The total content of the C8 compounds in the sample B (11.40 µg/g) was significantly higher than those in the sample A (4.52 µg/g) and sample C (3.84 µg/g) (*p* < 0.05), especially 1-octene-3-ol and 3-octanone, which were reported to contribute to mushroom-like odor [38]. The lesser extent of dryness exerted on sample B retained higher content of C8 compounds, compared with the sample A and C. The results were consistent with those obtained by GC-IMS. It revealed that the drying degree had a certain effect on the C8 compounds, and the lower the drying degree was, the less degradation C8 compounds was subjected to and the more mushroom-like odor was retained.

### 3.3. Aroma Properties of Shiitake Mushrooms under Different Hot Air Drying Conditions by DSA

**Figure 3:**
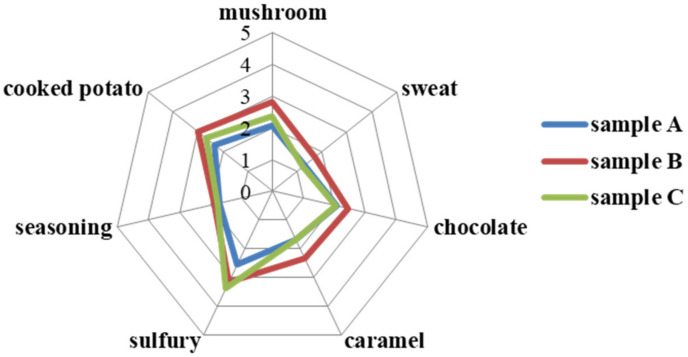
Radar chart of shiitake mushrooms with different hot-air-drying conditions by descriptive sensory analysis (DSA).

Seven aroma descriptors, including mushroom-like, chocolate-like, sulfury, caramel, sweat, seasoning-like and cooked potato-like odors, were determined as the main aroma properties of dried shiitake mushrooms in this study. Samples under the three different hot air-drying conditions were analyzed by DSA and the aroma profiles were plotted in Figure 3. It could be seen that sulfury attribute was the most outstanding aroma characteristic with the highest score among the seven aroma properties in the three samples. The unique sulfury flavor of dried mushrooms is mainly due to the activation of enzymes during the drying process, which act on lentinic acid to produce sulfur-containing heterocyclic compounds [15]. Sample A showed the weaker sulfury attribute than sample B and C (*p* < 0.05). This was consistent with the concentration of sulfur compounds by GC-MS analysis. With respect to the other six aroma properties, samples B always showed higher intensities than sample A and C (*p* < 0.05). These results showed that sample B with lesser dryness degree and higher moisture content had more obvious aroma characteristics and stronger odor intensity. It might be due to the fact that some flavor components were oxidized and degraded into other volatiles or non-volatile compounds during the later drying stage, which led to the decrease of the overall aroma intensity of shiitake mushrooms [40]. This indicated that drying degree had a noticeable influence on the aroma profile of shiitake mushrooms.

### 3.4. Discrimination Analysis of Shiitake Mushrooms under Different Hot Air Drying Conditions by Advanced Chemometric Methods

#### 3.4.1. Comparison Analysis of Shiitake Mushrooms Based on Volatile Fingerprints by PCA and FSA

**Figure 4:**
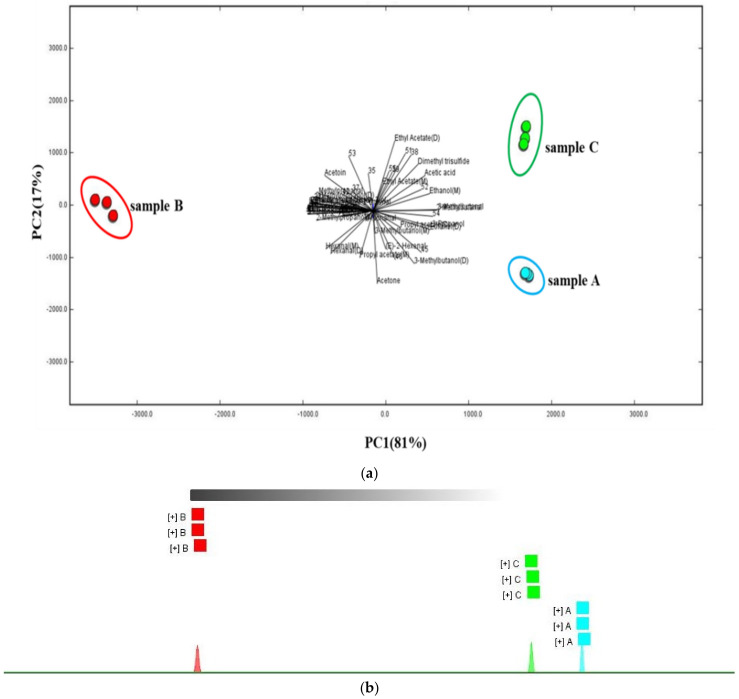
Principal component analysis (PCA) linked loading plots (**a**) and fingerprint similarity analysis (FSA) (**b**) of shiitake mushrooms under different hot air-drying conditions.

The PCA evaluated the regularity and difference among samples and the contribution of principal components by determining principal component factors instead of complex variables in the original samples [25,41]. Figure 4a demonstrated the score plot of PCA analysis. The first and second principal components (PC1 and PC2) represented 81% and 17%, respectively, and added up to 98% of the total variables. This indicated that the PCA could represent most information of the samples. The PCA obtained by the dynamic PCA module has also been successfully applied in the discrimination analysis in studies of kiwifruits [21], oil [42], shrimp [43], and pufferfish [44], based on the volatile fingerprints obtained by GC-IMS. As shown in Figure 4a, the sample A and C were at the right side. They were hardly separated along the PC1, but could be separated a little along the PC2. Sample B were at the left side and could be separated evidently from sample A and C, especially along the PC1. This illustrated that sample A and C were similar, compared with sample B. As shown by loading plots, ethyl acetate and dimethyl trisulfide was in close relationship with sample C. This may be due to the fact that drying contributed to the formation of dimethyl trisulfide [15,16]. Additionally, 3-methy-1-butanol was close to sample A and hexanal was connected with sample B. This was illustrated by the fact that 3-methy-1-butanol and hexanal stood out in sample A and B, respectively. These were consisted with information shown in the gallery plot. In addition, the Figure 4b showed that the three samples could be clearly divided into three categories, indicating their dissimilarities. But among the three samples, close distances between sample A and C and long distances between sample B and sample A and C were observed. It demonstrated that sample A and C were similar and sample B was more different. The results observed by the FSA were consistent with the PCA.

The two advanced chemometric methods both demonstrated the more similarities of the samples that were processed at different drying rate and the same drying degree, and more dissimilarities of the samples that were processed at the same drying rate and different drying degree. And this further revealed that flavor characteristics were influenced more by drying degree than by drying rate.

#### 3.4.2. Comparison Analysis of Shiitake Mushrooms Based on Volatile Compounds and Aroma Properties by PLSR

**Figure 5:**
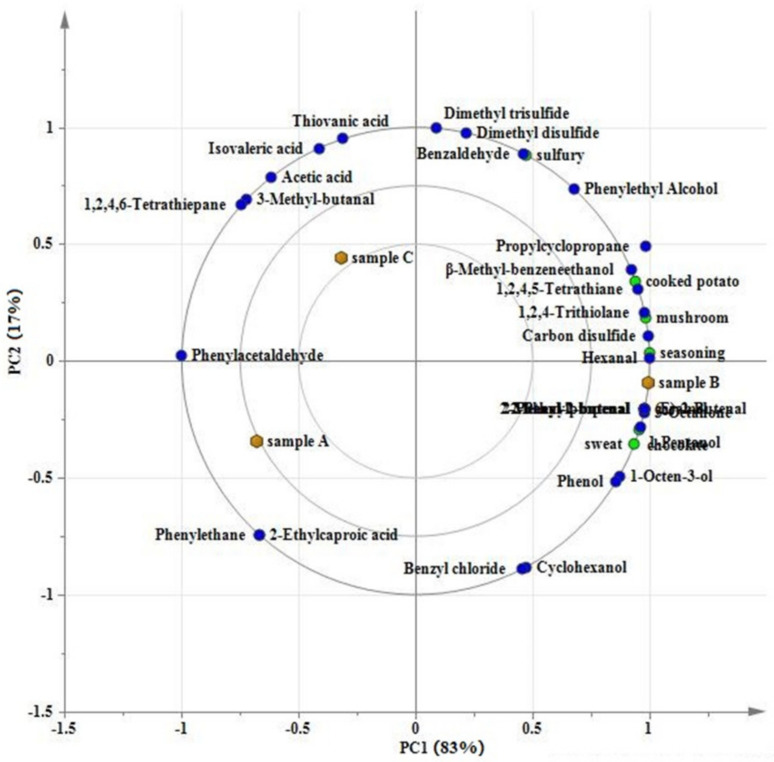
The partial least squares regression (PLSR) analysis between volatile compounds and aroma properties of shiitake mushrooms.

PLSR was utilized to show the characteristic of samples and relationship among the samples, volatile compounds and sensory properties based on the results of GC-MS and DSA. It has been successfully applied in volatile characteristic and comparison analysis of the shiitake mushrooms processed by three drying technologies [14] and three kinds of kiwifruits [21], also based on the volatile compounds obtained by GC-MS and sensory properties by DSA. In the PLSR analysis, X variable was designed as aroma properties and Y variable was designed as volatile compounds (RMSE 0.7396, R^2^ 0.9931). As shown in Figure 5, the PLSR provided a two-factor model, in which the inner, middle, and outer ellipses represented 50%, 70% and 100% of the interpreted variance, respectively. Most of the volatile compounds and sensory properties were placed between the inner and outer ellipses, suggesting that the model could be used to accurately account for these variables [21].

It could be seen from Figure 5 that the PC1 was the most important components and accounted for 83%. The sample A and sample C had a small distance along the PC1 and were both on the left side, whereas sample B was on the right side and away from sample A and C. This demonstrated that the sample B was different from sample A and C, with respect to aroma profile consisting of volatile compounds and aroma properties.

As shown in Figure 5, sulfury attribute was near to the sample C and the sample B, and far from the sample A. It illustrated that the sample C and sample B had the remarkable sulfur odor, and on the contrary the sample A had the weak sulfur odor. Additionally, the other 6 aroma attributes, including cooked potato, mushroom, seasoning, chocolate, caramel and sweat, were close to the sample B. It illustrated that the sample B was the most prominent in these aroma properties among the three samples. Furthermore, among the six aroma attributes, cooked potato, mushroom, and seasoning were nearer to the sample C than the sample A, no matter along the PC1 or PC2. This stated that these odors were more intense in the sample C than those in the sample A. It is noticeable that these results obtained by PLSR were consistent with those by DSA, further elucidating that the PLSR analysis was rational and effective in this study.

Moreover, it was shown in Figure 5 that 3-octanone, (E)-2-butenal, 2-methyl-2-butenal, 2-phenylpropenal, 2-phenyl-2-butenal, hexanal, 1-pentanol, carbon disulfide, 1,2,4-trithiolane, 1,2,4,5-tetrathiane, 1-octen-3-ol, phenol, and β-methyl-benzeneethanol, were closely linked to the sample B. As shown in Table 2, these compounds were only detected in the sample B or had significant higher concentrations than those in sample A and sample C. This gave the reason to their close relationship with the sample B. It has been reported that ketones, alcohols and aldehydes have been reported as the main volatile compounds in fresh shiitake mushrooms [10,45]. But drying dehydration could result in the loss of ketones, alcohols and aldehydes [46]. Ketones, alcohols and aldehydes were easily destroyed through a series of enzymatic and non-enzymatic reactions during drying [15]. Thus, sample B with lower drying degree retained more ketone, alcohol and aldehyde compounds. 3-Methylbutanal, acetic acid, isovaleric acid, thiovanic acid, and 1,2,4,6-tetrathiepane were relatively close to the sample C, illustrating that they were the representative compounds of the sample C. It was consistent with the results of GC-MS analysis that the sample C had outstanding abundance in these compounds. Additionally, the sample A was associated with phenylethane and 2-ethylcaproic acid as shown in Figure 5, which were only detected in the sample A by GC-MS.

In addition, the relationship between aroma properties and volatile compounds also could be illustrated in Figure 5. Sulfury attribute was highly correlated with dimethyl disulfide and dimethyl trisulfide. These two compounds have been reported to be the key odorants in dried shiitake mushrooms [47]. Cooked potato attribute was positively associated with β-methyl-benzeneethanol and 1,2,4,5-tetrathiane. Mushroom-like odor was strongly associated with 1,2,4-trithiolane and carbon disulfide. Hexanal was regarded as the main contributor to seasoning attribute. 1-Pentanol had a good correlation with chocolate attribute. Caramel attribute was closely related to 3-octanone, 2-methyl-2-butenal, (E)-2-butenal, 2-phenylpropenal and 2-phenyl-2-butenal. These volatile flavor substances which were strongly associated with aroma attributes might impact the overall aroma of shiitake mushrooms markedly.

## 4. Conclusions

The aroma fingerprints and discrimination analysis of shiitake mushrooms under different drying conditions were performed by GC-IMS, GC-MS, and DSA with advanced chemometric methods in this study. The sample B had the same drying rate as sample C and their rate was lower than sample A. The sample A had the same drying degree as sample C and their degree was larger than sample B. Higher signal intensities of volatile compounds of sample B were observed through the volatile fingerprints by GC-IMS. As the volatile profile shown, sample B not only had more varieties of volatile compounds than sample A and C, but also had significant higher abundance in volatile compounds. Furthermore, more obvious aroma characteristics and stronger odor intensity in mushroom-like, chocolate-like, sulfury, caramel, sweat, seasoning-like and cooked potato-like odors were detected in the sample B by DSA. Thus, the volatile flavor was enriched in the sample with lower degree of dryness. Moreover, the sample B could be separated evidently from sample A and C through PCA, PLSR, and FSA. The advanced chemometric methods demonstrated the more similarities of the samples at different drying rate and more dissimilarities of the samples at different drying degree. This study revealed that drying degree exerted more influence on the volatile flavor quality of dried shiitake mushrooms than the drying rate. The study will provide foundation for the flavor formation and variation mechanism study and establish a comprehensive and objective method for flavor analysis. In the future study, the possible mechanisms related to the dissimilarities of shiitake mushrooms processed by different conditions will be revealed. This will promote the flavor enhancing in mushroom products and benefit the mushroom production industry.

## Figures and Tables

**Table 1:** Identification of volatile flavor compounds in shiitake mushrooms with different hot-air-drying conditions by GC-IMS.

No.	RI	Compound	CAS Number	Formula	Comment
1	990.8	1-Octen-3-ol	3391-86-4	C8H16O	
2	991.0	3-Octanone	106-68-3	C8H16O	Monomer
3	991.8	3-Octanone	106-68-3	C8H16O	Dimer
4	975.3	Benzaldehyde	100-52-7	C7H6O	Monomer
5	975.3	Benzaldehyde	100-52-7	C7H6O	Dimer
6	973.2	Dimethyl trisulfide	3658-80-8	C2H6S3	
7	794.6	Hexanal	66-25-1	C6H12O	Monomer
8	795.4	Hexanal	66-25-1	C6H12O	Dimer
9	742.3	(E)-2-Pentenal	1576-87-0	C5H8O	
10	739.5	3-Methyl-1-butanol	123-51-3	C5H12O	Monomer
11	740.2	3-Methyl-1-butanol	123-51-3	C5H12O	Dimer
12	709.7	Propyl acetate	109-60-4	C5H10O2	Monomer
13	707.4	Propyl acetate	109-60-4	C5H10O2	Dimer
14	603.6	Ethyl acetate	141-78-6	C4H8O2	Monomer
15	604.1	Ethyl acetate	141-78-6	C4H8O2	Dimer
16	559.5	2-Methylpropanal	78-84-2	C4H8O	
17	583.5	Acetic acid	64-19-7	C2H4O2	
18	507.6	2-Propanol	67-63-0	C3H8O	
19	502.6	Acetone	67-64-1	C3H6O	
20	476.2	Ethanol	64-17-5	C2H6O	Monomer
21	480.4	Ethanol	64-17-5	C2H6O	Dimer
22	724.2	Acetoin	513-86-0	C4H8O2	
23	624.9	2-Methyl-1-propanol	78-83-1	C4H10O	Monomer
24	625.2	2-Methyl-1-propanol	78-83-1	C4H10O	Dimer
25	1102.4	1-Nonanal	124-19-6	C9H18O	
26	1067.5	(E)-2-Octenal	2548-87-0	C8H14O	
27	1065.2	Phenylacetaldehyde	122-78-1	C8H8O	
28	903.5	Heptanal	111-71-7	C7H14O	
29	862.0	(E)-2-Hexenal	6728-26-3	C6H10O	
30	588.7	Butanal	123-72-8	C4H8O	
31	662.4	2-Methylbutanal	96-17-3	C5H10O	
32	645.8	3-Methylbutanal	590-86-3	C5H10O	

RI, retention index; CAS, chemical abstracts service.

**Table 2:** Identification of volatile compounds of shiitake mushrooms with different hot-air-drying conditions.

No.	RI	Compound	CAS Number	ID	Concentrations (µg/g)		
					Sample A	Sample B	Sample C
Sulfur compounds (7)							
1	735	Carbon disulfide	75-15-0	RI, MS	6.17 ± 1.34 b	9.34 ± 0.29 a	7.18 ± 0.34 b
4	1083	Dimethyl disulfide	624-92-0	RI,MS,S	0.71 ± 0.02 c	1.07 ± 0.10 b	1.42 ± 0.09 a
9	1252	Thiovanic acid	68-11-1	RI,MS	ND	ND	0.20 ± 0.03 a
12	1350	Dimethyl trisulfide	3658-80-8	RI,MS,S	2.06 ± 0.34 c	3.37 ± 0.72 b	5.37 ± 0.29 a
20	1709	1,2,4-Trithiolane	289-16-7	RI,MS	12.77 ± 0.15 c	20.35 ± 2.56 a	15.84 ± 0.63 b
27	2196	1,2,4,5-Tetrathiane	291-22-5	RI,MS	2.09 ± 0.33 b	3.31 ± 0.8 a	2.70 ± 0.11 ab
28	2498	1,2,4,6-Tetrathiepane	292-45-5	RI,MS	0.83 ± 0.12 a	0.74 ± 0.00 a	0.92 ± 0.10 a
Total content of sulfur compounds					24.63 ± 2.00 b	38.18 ± 3.78 a	33.63 ± 0.89 a
C8 compounds (3)							
10	1260	3-Octanone	106-68-3	RI,MS,S	0.10 ± 0.03 b	3.55 ± 0.33 a	0.05 ± 0.01 b
15	1446	1-Octen-3-ol	3391-86-4	RI,MS,S	1.59 ± 0.27 b	4.38 ± 0.33 a	0.34 ± 0.04 c
22	1900	Phenylethyl alcohol	60-12-8	RI,MS,S	2.83 ± 0.49 a	3.47 ± 0.35 a	3.46 ± 0.22 a
Total content of C8 compounds					4.52 ± 0.75 b	11.40 ± 0.34 a	3.84 ± 0.21 b
Miscellaneous (18)							
2	934	3-Methylbutanal	590-86-3	RI,MS,S	1.86 ± 0.03 ab	1.59 ± 0.10 b	2.16 ± 0.36 a
3	1056	(E)-2-Butenal	123-73-9	RI,MS	ND	1.82 ± 0.40 a	ND
5	1103	Hexanal	66-25-1	RI,MS	0.17 ± 0.06 b	0.30 ± 0.01 a	0.20 ± 0.01 b
6	1109	2-Methyl-2-butenal	497-03-0	RI,MS	ND	0.70 ± 0.01 a	ND
7	1134	Phenylethane	100-41-4	RI,MS	0.95 ± 0.13 a	ND	ND
8	1214	1-Pentanol	71-41-0	RI,MS	0.41 ± 0.03 b	1.15 ± 0.19 a	0.34 ± 0.03 b
11	1344	1-Cyclopropylpropane	2415-72-7	RI,MS	0.54 ± 0.22 a	0.67 ± 0.05 a	ND
13	1385	Cyclohexanol	108-93-0	RI,MS	1.33 ± 0.14 a	1.36 ± 0.15 a	1.20 ± 0.11 a
14	1441	Acetic acid	64-19-7	RI,MS,S	0.15 ± 0.10 b	ND	0.43 ± 0.01 a
16	1489	Benzyl chloride	100-44-7	RI,MS	0.20 ± 0.06 a	0.22 ± 0.01 a	0.10 ± 0.01 c
17	1501	Benzaldehyde	100-52-7	RI,MS,S	2.08 ± 0.29 c	5.46 ± 0.76 b	6.66 ± 0.62 a
18	1638	Phenylacetaldehyde	122-78-1	RI,MS,S	1.65 ± 0.68 a	1.04 ± 0.43 a	1.53 ± 0.21 a
19	1659	Isovaleric acid	503-74-2	RI,MS,S	1.21 ± 0.03 b	1.06 ± 0.14 b	2.29 ± 0.35 a
21	1789	2-Phenylpropenal	4432-63-7	RI,MS	ND	0.26 ± 0.05 a	ND
23	1921	β-Methylbenzeneethanol	1123-85-9	RI,MS	0.94 ± 0.16 c	1.83 ± 0.17 a	1.45 ± 0.22 b
24	1926	2-Phenyl-2-butenal	4411-89-6	RI,MS	ND	2.95 ± 0.50 a	ND
25	1952	2-Ethylcaproic acid	149-57-5	RI,MS	0.33 ± 0.03 a	ND	ND
26	1997	Phenol	108-95-2	RI,MS	0.06 ± 0.01 b	0.18 ± 0.05 a	ND
Total content of miscellaneous					11.87 ± 1.18 c	20.60 ± 1.78 a	16.36 ± 0.57 b
Total content of volatile compounds					41.02 ± 3.67 c	70.18 ± 5.71 a	53.83 ± 1.57 b

All data were the means ± SD, *n* = 3; means with the same letter are not significantly different between different samples (*p* < 0.05). The No. referred to the values of RI; CAS, chemical abstracts service; ID, identification methods; RI, retention index, MS, mass spectra; S, standards; ND, not detected.
